# COVID-19 image classification using deep features and fractional-order marine predators algorithm

**DOI:** 10.1038/s41598-020-71294-2

**Published:** 2020-09-21

**Authors:** Ahmed T. Sahlol, Dalia Yousri, Ahmed A. Ewees, Mohammed A. A. Al-qaness, Robertas Damasevicius, Mohamed Abd Elaziz

**Affiliations:** 1 Computer Department, Damietta University, Damietta, Egypt; 2 Electrical Engineering Department, Faculty of Engineering, Fayoum University, Fayoum, Egypt; 3 State Key Laboratory for Information Engineering in Surveying, Mapping, and Remote Sensing, Wuhan University, Wuhan, China; 4 Department of Applied Informatics, Vytautas Magnus University, Kaunas, Lithuania; 5 Department of Mathematics, Faculty of Science, Zagazig University, Zagazig, Egypt; 6 School of Computer Science and Robotics, Tomsk Polytechnic University, Tomsk, Russia

**Keywords:** Computational models, Image processing, Machine learning

## Abstract

Currently, we witness the severe spread of the pandemic of the new Corona virus, COVID-19, which causes dangerous symptoms to humans and animals, its complications may lead to death. Although convolutional neural networks (CNNs) is considered the current state-of-the-art image classification technique, it needs massive computational cost for deployment and training. In this paper, we propose an improved hybrid classification approach for COVID-19 images by combining the strengths of CNNs (using a powerful architecture called Inception) to extract features and a swarm-based feature selection algorithm (Marine Predators Algorithm) to select the most relevant features. A combination of fractional-order and marine predators algorithm (FO-MPA) is considered an integration among a robust tool in mathematics named fractional-order calculus (FO). The proposed approach was evaluated on two public COVID-19 X-ray datasets which achieves both high performance and reduction of computational complexity. The two datasets consist of X-ray COVID-19 images by international Cardiothoracic radiologist, researchers and others published on Kaggle. The proposed approach selected successfully 130 and 86 out of 51 K features extracted by inception from dataset 1 and dataset 2, while improving classification accuracy at the same time. The results are the best achieved on these datasets when compared to a set of recent feature selection algorithms. By achieving 98.7%, 98.2% and 99.6%, 99% of classification accuracy and F-Score for dataset 1 and dataset 2, respectively, the proposed approach outperforms several CNNs and all recent works on COVID-19 images.

## Introduction

Currently, a new coronavirus, called COVID-19, has spread to many countries, with over two million infected people or so-called confirmed cases. Also, it has killed more than 376,000 (up to 2 June 2020) [Coronavirus disease (COVID-2019) situation reports: (https://www.who.int/emergencies/diseases/novel-coronavirus-2019/situation-reports/)]. The family of coronaviruses is considered serious pathogens for people because they infect respiratory, hepatic, gastrointestinal, and neurologic diseases. They are distributed among people, bats, mice, birds, livestock, and other animals^1,2^. In the last two decades, two famous types of coronaviruses SARS-CoV and MERS-CoV had been reported in 2003 and 2012, in China, and Saudi Arabia, respectively^3^. Although outbreaks of SARS and MERS had confirmed human to human transmission^3^, they had not the same spread speed and infection power of the new coronavirus (COVID-19).

For diagnosing COVID-19, the RT-PCR (real-time polymerase chain reaction) is a standard diagnostic test, but, it can be considered as a time-consuming test, more so, it also suffers from false negative diagnosing^4^. However, using medical imaging, chest CT, and chest X-ray scan can play a critical role in COVID-19 diagnosis.

Medical imaging techniques are very important for diagnosing diseases. Image segmentation is a necessary image processing task that applied to discriminate region of interests (ROIs) from the area of outsides. Also, image segmentation can extract critical features, including the shape of tissues, and texture^5,6^.

In general, feature selection (FS) methods are widely employed in various applications of medical imaging applications. For example, Lambin et al.^7^ proposed an efficient approach called Radiomics to extract medical image features. They showed that analyzing image features resulted in more information that improved medical imaging. Chong et al.^8^ proposed an FS model, called Robustness-Driven FS (RDFS) to select futures from lung CT images to classify the patterns of fibrotic interstitial lung diseases. They applied the SVM classifier with and without RDFS. The evaluation showed that the RDFS improved SVM robustness against reconstruction kernel and slice thickness. In^9^, to classify ultrasound medical images, the authors used distance-based FS methods and a Fuzzy Support Vector Machine (FSVM). Moreover, a multi-objective genetic algorithm was applied to search for the optimal features subset.

More so, a combination of partial differential equations and deep learning was applied for medical image classification by^10^. They employed partial differential equations for extracting texture features of medical images. Acharya et al.^11^ applied different FS methods to classify Alzheimer’s disease using MRI images. The Shearlet transform FS method showed better performances compared to several FS methods. Also, in^12^, an Fs method based on SVM was proposed to detect Alzheimer’s disease from SPECT images. Duan et al.^13^ applied the Gaussian mixture model (GMM) to extract features from pulmonary nodules from CT images. The optimum path forest (OPF) classifier was applied to classify pulmonary nodules based on CT images. In^14^, the authors proposed an FS method based on a convolutional neural network (CNN) to detect pneumonia from lung X-ray images.

Afzali et al.^15^ proposed an FS method based on principal component analysis and contour-based shape descriptors to detect Tuberculosis from lung X-Ray Images. They used K-Nearest Neighbor (kNN) to classify x-ray images collected from Montgomery dataset, and it showed good performances. Zhang et al.^16^ proposed a kernel feature selection method to segment brain tumors from MRI images. They applied the SVM classifier for new MRI images to segment brain tumors, automatically. To segment brain tissues from MRI images, Kong et al.^17^ proposed an FS method using two methods, called a discriminative clustering method and the information theoretic discriminative segmentation. Harikumar et al.^18^ proposed an FS method based on wavelets to classify normality or abnormality of different types of medical images, such as CT, MRI, ultrasound, and mammographic images. It can be concluded that FS methods have proven their advantages in different medical imaging applications^19^.

Furthermore, deep learning using CNN is considered one of the best choices in medical imaging applications^20^, especially classification. CNNs are more appropriate for large datasets. Also, they require a lot of computational resources (memory & storage) for building & training. In some cases (as exists in this work), the dataset is limited, so it is not sufficient for building & training a CNN. In such a case, in order to get the advantage of the power of CNN and also, transfer learning can be applied to minimize the computational costs^21,22^. In transfer learning, a CNN which was previously trained on a large & diverse image dataset can be applied to perform a specific classification task by^23^. Therefore, several pre-trained models have won many international image classification competitions such as VGGNet^24^, Resnet^25^, Nasnet^26^, Mobilenet^27^, Inception^28^ and Xception^29^.

However, some of the extracted features by CNN might not be sufficient, which may affect negatively the quality of the classification images. Therefore, a feature selection technique can be applied to perform this task by removing those irrelevant features. Among the FS methods, the metaheuristic techniques have been established their performance overall other FS methods when applied to classify medical images. For example, Da Silva et al.^30^ used the genetic algorithm (GA) to develop feature selection methods for ranking the quality of medical images. They used different images of lung nodules and breast to evaluate their FS methods. Evaluation outcomes showed that GA based FS methods outperformed traditional approaches, such as filter based FS and traditional wrapper methods. Johnson et al.^31^ applied the flower pollination algorithm (FPA) to select features from CT images of the lung, to detect lung cancers. They also used the SVM to classify lung CT images. The evaluation confirmed that FPA based FS enhanced classification accuracy. kharrat and Mahmoud^32^proposed an FS method based on a hybrid of Simulated Annealing (SA) and GA to classify brain tumors using MRI. The combination of SA and GA showed better performances than the original SA and GA. Narayanan et al.^33^ proposed a fuzzy particle swarm optimization (PSO) as an FS method to enhance the classification of CT images of emphysema. They applied a fuzzy decision tree classifier, and they found that fuzzy PSO improved the classification accuracy. Li et al.^34^ proposed a self-adaptive bat algorithm (BA) to address two problems in lung X-ray images, rebalancing, and feature selection. They compared the BA to PSO, and the comparison outcomes showed that BA had better performance. Dhanachandra and Chanu^35^ proposed a hybrid method of dynamic PSO and fuzzy c-means to segment two types of medical images, MRI and synthetic images. They concluded that the hybrid method outperformed original fuzzy c-means, and it had less sensitive to noises. Li et al.^36^ proposed an FS method using a discrete artificial bee colony (ABC) to improve the classification of Parkinson’s disease. The evaluation outcomes demonstrate that ABC enhanced precision, and also it reduced the size of the features.

In this paper, we proposed a novel COVID-19 X-ray classification approach, which combines a CNN as a sufficient tool to extract features from COVID-19 X-ray images. Then, using an enhanced version of Marine Predators Algorithm to select only relevant features. In general, MPA is a meta-heuristic technique that simulates the behavior of the prey and predator in nature^37^. This algorithm is tested over a global optimization problem. However, it has some limitations that affect its quality. In addition, up to our knowledge, MPA has not applied to any real applications yet. So, based on this motivation, we apply MPA as a feature selector from deep features that produced from CNN (largely redundant), which, accordingly minimize capacity and resources consumption and can improve the classification of COVID-19 X-ray images.

In this work, the MPA is enhanced by fractional calculus memory feature, as a result, Fractional-order Marine Predators Algorithm (FO-MPA) is introduced. Moreover, the Weibull distribution employed to modify the exploration function.

The proposed COVID-19 X-ray classification approach starts by applying a CNN (especially, a powerful architecture called Inception which pre-trained on Imagnet dataset) to extract the discriminant features from raw images (with no pre-processing or segmentation) from the dataset that contains positive and negative COVID-19 images. Then, applying the FO-MPA to select the relevant features from the images. This task is achieved by FO-MPA which randomly generates a set of solutions, each of them represents a subset of potential features. The next process is to compute the performance of each solution using fitness value and determine which one is the best solution. Thereafter, the FO-MPA parameters are applied to update the solutions of the current population. The updating operation repeated until reaching the stop condition. Then the best solutions are reached which determine the optimal/relevant features that should be used to address the desired output via several performance measures. Inspired by our recent work^38^, where VGG-19 besides statistically enhanced Salp Swarm Algorithm was applied to select the best features for White Blood Cell Leukaemia classification. Also, other recent published works^39^, who combined a CNN architecture with Weighted Symmetric Uncertainty (WSU) to select optimal features for traffic classification. It is obvious that such a combination between deep features and a feature selection algorithm can be efficient in several image classification tasks.

The main contributions of this study are elaborated as follows:

1. Propose an efficient hybrid classification approach for COVID-19 using a combination of CNN and an improved swarm-based feature selection algorithm. This combination should achieve two main targets; high performance and resource consumption, storage capacity which consequently minimize processing time.
2. Propose a novel robust optimizer called Fractional-order Marine Predators Algorithm (FO-MPA) to select efficiently the huge feature vector produced from the CNN.
3. Test the proposed Inception Fractional-order Marine Predators Algorithm (IFM) approach on two publicity available datasets contain a number of positive negative chest X-ray scan images of COVID-19.
4. Evaluate the proposed approach by performing extensive comparisons to several state-of-art feature selection algorithms, most recent CNN architectures and most recent relevant works and existing classification methods of COVID-19 images.

We do not present a usable clinical tool for COVID-19 diagnosis, but offer a new, efficient approach to optimize deep learning-based architectures for medical image classification purposes. Such methods might play a significant role as a computer-aided tool for image-based clinical diagnosis soon. Remainder sections are organized as follows: “Material and methods” section presents the methodology and the techniques used in this work including model structure and description. The experimental results and comparisons with other works are presented in “Results and discussion” section, while they are discussed in “Discussion” section Finally, the conclusion is described in “Conclusion” section.

## Material and methods

### Features extraction using convolutional neural networks

In this paper, we apply a convolutional neural network (CNN) to extract features from COVID-19 X-Ray images. We adopt a special type of CNN called a pre-trained model where the network is previously trained on the ImageNet dataset, which contains millions of variety of images (animal, plants, transports, objects,..) on 1000 classe categories. So, transfer learning is applied by transferring weights that were already learned and reserved into the structure of the pre-trained model, such as Inception, in this paper.

**Figure 1:**
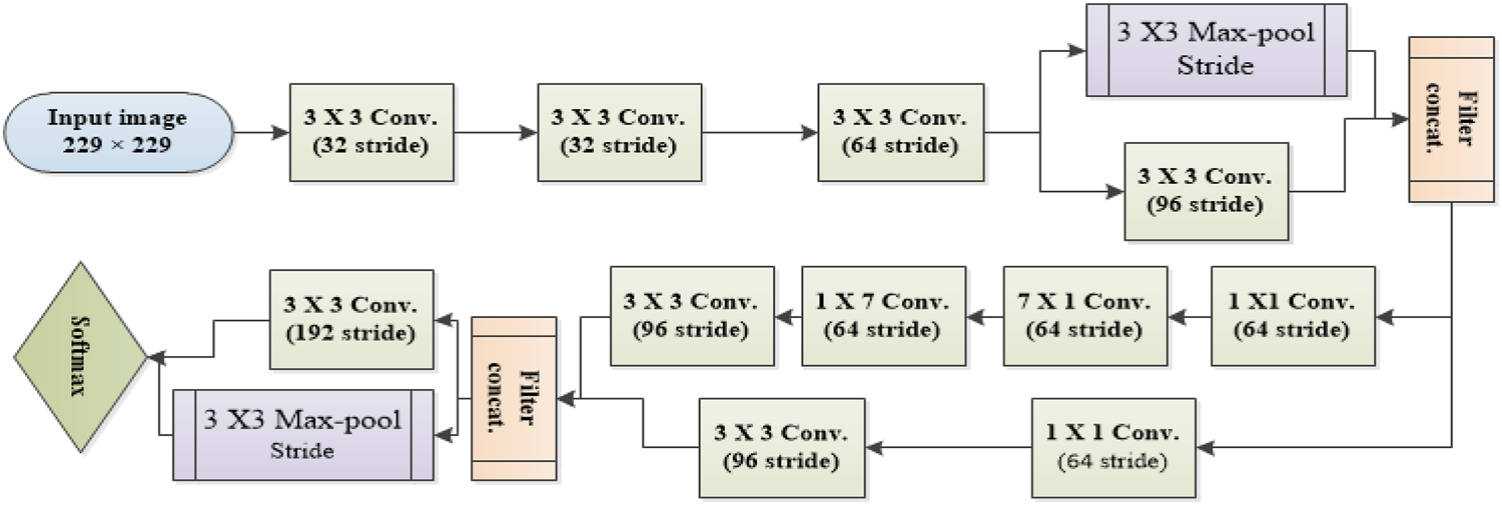
Overview of inception.

In Inception, there are different sizes scales convolutions (conv.), such as $5\times 5$, $3 \times 3$, $1 \times 1$. For instance,$1\times 1$ conv. is applied before larger sized kernels are applied to reduce the dimension of the channels, which accordingly, reduces the computation cost. Pool layers are used mainly to reduce the input’s size, which accelerates the computation as well. So, for a $4 \times 4$ matrix, will result in $2 \times 2$ matrix after applying max pooling. There are three main parameters for pooling, Filter size, Stride, and Max pool. In this paper, filters of size 2, besides a stride of 2 and $2 \times 2$ as Max pool, were adopted. Inception architecture is described in Fig. 1.

The main purpose of Conv. layers is to extract features from input images. In this paper, different Conv. Layers are applied to extract different types of features such as edges, texture, colors, and high-lighted patterns from the images. The combination of Conv. and pool layers, three fully connected layers, the last one performs classification. The Softmax activation function is used for this purpose because the output should be binary (positive COVID-19 negative COVID-19). Inception’s layer details and layer parameters of are given in Table 1.

**Table 1:** Layer parameters of Inception.

Layer number	Layer type	Output Shape	Number of trainable parameters
1	conv2d_1	(114, 114, 324)	864
|	|	|	|
10	conv2d_1_0	(26, 26, 96)	55296
|	|	|	|
20	conv2d_2_0	(26, 26, 64)	18432
|	|	|	|
30	conv2d_3_0	(12, 12, 96)	82944
|	|	|	|
40	conv2d_4_0	(12, 12, 192)	147456
|	|	|	|
50	conv2d_5_0	(12, 12, 192)	147456
|	|	|	|
60	conv2d_6_0	(12, 12, 192)	147456
|	|	|	|
70	conv2d_7_0	(12, 12, 192)	147456
|	|	|	|
80	conv2d_8_0	(5, 5, 384)	442368
|	|	|	|
94	conv2d_9_4	(5, 5, 192)	393216
|	|	|	|
159	mixed10 (Concatenate)	(5, 5, 2048)	0

As seen in Table 1, we keep the last concatenation layer which contains the extracted features, so we removed the top layers such as the Flatten, Drop out and the Dense layers which the later performs classification (named as FC layer). We have used RMSprop optimizer for weight updates, cross entropy loss function and selected learning rate as 0.0001.

In this paper, Inception is applied as a feature extractor, where the input image shape is (229, 229, 3). Since its structure consists of some parallel paths, all the paths use padding of 1 pixel to preserve the same height & width for the inputs and the outputs.

One of the drawbacks of pre-trained models, such as Inception, is that its architecture required large memory requirements as well as storage capacity (92 M.B), which makes deployment exhausting and a tiresome task. The shape of the output from the Inception is (5, 5, 2048), which represents a feature vector of size 51200. So some statistical operations have been added to exclude irrelevant and noisy features, and by making it more computationally efficient and stable, they are summarized as follows:

- Chi-square is applied to remove the features which have a high correlation values by computing the dependence between them. It is calculated between each feature for all classes, as in Eq. (1):
  1 $$\begin{aligned} \chi ^2=\sum _{k=1}^{n} \frac{(O_k - E_k)^2}{E_k} \end{aligned}$$
  where $O_k$ and $E_k$ refer to the actual and the expected feature value, respectively. In this paper, after applying Chi-square, the feature vector is minimized for both datasets from 51200 to 2000.
- Tree based classifier are the most popular method to calculate feature importance to improve the classification since they have high accuracy, robustness, and simple^38^. For each decision tree, node importance is calculated using Gini importance, Eq. (2) calculated two child nodes.
  2 $$\begin{aligned} ni_{j}=w_{j}C_{j}-w_{left(j)}C_{left(j)}-w_{right(j)}C_{right(j)} \end{aligned}$$
  where $ni_{j}$ is the importance of node j, while $w_{j}$ refers to the weighted number of samples reaches the node j, also $C_{j}$ determines the impurity value of node j. left(j) and right(j) are the child nodes from the left split and the right split on node j, respectively. In Eq. (3), the importance of each feature is then calculated.
  3 $$\begin{aligned} fi_{i}=\frac{\sum _{j:node \mathbf \ {j} \ splits \ on \ feature \ i}ni_{j}}{\sum _{{k}\in all \ nodes }ni_{k}} \end{aligned}$$
  where $fi_{i}$ represents the importance of feature I, while $ni_{j}$ refers to the importance of node j. In order to normalize the values between 0 and 1 by dividing by the sum of all feature importance values, as in Eq. (4).
  4 $$\begin{aligned} normfi_{i}=\frac{fi_{i}}{\sum _{{j}\in all \ nodes }fi_{j}} \end{aligned}$$
  Finally, the sum of the feature’s importance value on each tree is calculated then divided by the total number of trees as in Eq. (5).
  5 $$\begin{aligned} REfi_{i}=\frac{\sum _{j \in all trees} normfi_{ij}}{T} \end{aligned}$$
  where $REfi_{i}$ represents the importance of feature i that were calculated from all trees, where $normfi_{ij}$ is the normalized feature importance for feature i in tree j, also T is the total number of trees.
  After applying this technique, the feature vector is minimized from 2000 to 459 and from 2000 to 462 for Dataset1 and Dataset 2, respectively.

### Feature selection by fractional-order calculus with Marine Predators Algorithm (FO-MPA)

#### Fractional calculus (FC)

Fractional-order calculus (FC) gains the interest of many researchers in different fields not only in the modeling sectors but also in developing the optimization algorithms. The memory properties of Fc calculus makes it applicable to the fields that required non-locality and memory effect. FC provides a clear interpretation of the memory and hereditary features of the process. Accordingly, the FC is an efficient tool for enhancing the performance of the meta-heuristic algorithms by considering the memory perspective during updating the solutions. One from the well-know definitions of FC is the Grunwald-Letnikov (GL), which can be mathematically formulated as below^40^:

6 $$\begin{aligned} D^{\delta }(U(t))=\lim \limits _{h \rightarrow 0} \frac{1}{h^\delta } \sum _{k=0}^{\infty }(-1)^{k} \begin{pmatrix} \delta \\ k\end{pmatrix} U(t-kh), \end{aligned}$$

where

7 $$\begin{aligned} \begin{pmatrix} \delta \\ k \end{pmatrix}= \frac{\Gamma (\delta +1)}{\Gamma (k+1)\Gamma (\delta -k+1)}= \frac{\delta (\delta -1)(\delta -2)\ldots (\delta -k+1)}{k!}, \end{aligned}$$

where $D^{\delta }(U(t))$ refers to the GL fractional derivative of order $\delta$. $\Gamma (t)$ indicates gamma function.

The GL in the discrete-time form can be modeled as below:

8 $$\begin{aligned} D^{\delta }[U(t)]=\frac{1}{T^\delta }\sum _{k=0}^{m} \frac{(-1)^k\Gamma (\delta +1)U(t-kT)}{\Gamma (k+1)\Gamma (\delta -k+1)} \end{aligned}$$

where *T* is the sampling period, and *m* is the length of the memory terms (memory window). The $\delta$ symbol refers to the derivative order coefficient.

For the special case of $\delta = 1$, the definition of Eq. (8) can be remodeled as below:

9 $$\begin{aligned} D^1[U(t)]=U(t+1)-U(t) \end{aligned}$$

where $D^1[x(t)]$ represents the difference between the two followed events.

#### Marine Predators Algorithm

The Marine Predators Algorithm (MPA)is a recently developed meta-heuristic algorithm that emulates the relation among the prey and predator in nature^37^. MPA simulates the main aim for most creatures that is searching for their foods, where a predator contiguously searches for food as well as the prey. Inspired by this concept, Faramarzi et al.^37^ developed the MPA algorithm by considering both of a predator a prey as solutions. The MPA starts with the initialization phase and then passing by other three phases with respect to the rational velocity among the prey and the predator.

- Initialization phase: this phase devotes for providing a random set of solutions for both the prey and predator via the following formulas:
  10 $$\begin{aligned} U=Lower+rand_1\times (Upper - Lower ) \end{aligned}$$
  where the *Lower* and *Upper* are the lower and upper boundaries in the search space, $rand_1$ is a random vector $\in$ the interval of (0,1). According to the formula 10, the initial locations of the prey and predator can be defined as below:
  11 $$\begin{aligned} Elite=\left[ \begin{array}{cccc} U_{11}^1&{}U_{12}^1&{}\ldots &{}U_{1d}^1\\ U_{21}^1&{}U_{22}^1&{}\ldots &{}U_{2d}^1\\ \ldots &{}\ldots &{}\ldots &{}\ldots \\ U_{n1}^1&{}U_{n2}^1&{}\ldots &{}U_{nd}^1\\ \end{array}\right] , \, U=\left[ \begin{array}{cccc} U_{11}&{}U_{12}&{}\ldots &{}U_{1d}\\ U_{21}&{}U_{22}&{}\ldots &{}U_{2d}\\ \ldots &{}\ldots &{}\ldots &{}\ldots \\ U_{n1}&{}U_{n2}&{}\ldots &{}U_{nd}\\ \end{array}\right] , \, \end{aligned}$$
  where the Elite matrix refers to the fittest predators.
- Stage 1: After the initialization, the exploration phase is implemented to discover the search space. Therefore in MPA, for the first third of the total iterations, i.e., $\frac{1}{3}t_{max}$). Accordingly, the prey position is upgraded based the following equations.
  12 $$\begin{aligned} S_i&= {} R_B \bigotimes (Elite_i-R_B\bigotimes U_i), i=1,2,\ldots ,n \end{aligned}$$
  13 $$\begin{aligned} U_i&= {} U_i+P.R\bigotimes S_i \end{aligned}$$
  where $R\in [0,1]$ is a random vector drawn from a uniform distribution and $P=0.5$ is a constant number. The symbol $R_B$ refers to Brownian motion. $\bigotimes$ indicates the process of element-wise multiplications.
- Stage 2: The prey/predator in this stage begin exploiting the best location that detects for their foods. Stage 2 has been executed in the second third of the total number of iterations when $\frac{1}{3}t_{max}< t< \frac{2}{3}t_{max}$. Faramarzi et al.^37^ divided the agents for two halves and formulated Eqs. (14)–(15) to emulate the motion of the first half of the population (prey) and Eqs. (18)–(19) for the second half (predator) as represented below.
  14 $$\begin{aligned} S_i&= {} R_L \bigotimes (Elite_i-R_L\bigotimes U_i), i=1,2,\ldots ,n/2 \end{aligned}$$
  15 $$\begin{aligned} U_i&= {} U_i+P.R\bigotimes S_i \end{aligned}$$
  where $R_L$ has random numbers that follow Lévy distribution. Eq. (14)-(15) are implemented in the first half of the agents that represent the exploitation. While the second half of the agents perform the following equations.
  16 $$\begin{aligned} S_i&= {} R_B \bigotimes (R_B \bigotimes Elite_i- U_i), i=1,2,\ldots ,n/2 \end{aligned}$$
  17 $$\begin{aligned} U_i&= {} Elite_i+P.CF\bigotimes S_i,\, CF= \left( 1-\frac{t}{t_{max}} \right) ^{\left(2\frac{t}{t_{max}}\right) } \end{aligned}$$
  where *CF* is the parameter that controls the step size of movement for the predator.
- Stage 3: This stage executed on the last third of the iteration numbers ($t>\frac{2}{3}t_{max}$) where based on the following formula:
  18 $$\begin{aligned} S_i&= {} R_L \bigotimes (R_L \bigotimes Elite_i- U_i), i=1,2,\ldots ,n \end{aligned}$$
  19 $$\begin{aligned} U_i&= {} Elite_i+P.CF\bigotimes S_i,\, CF= \left( 1-\frac{t}{t_{max}}\right) ^{\left(2\frac{t}{t_{max}} \right) } \end{aligned}$$
- Eddy formation and Fish Aggregating Devices’ effect: Faramarzi et al.^37^ considered the external impacts from the environment, such as the eddy formation or Fish Aggregating Devices (FADs) effects to avoid the local optimum solutions. This stage can be mathematically implemented as below:
  20 $$\begin{aligned} U_i=\left\{ \begin{array}{ll} U_i+CF [U_{min}+R \bigotimes (U_{max}-U_{min})]\bigotimes W &{} r_5 < FAD \\ U_i+[FAD(1-r)+r](U_{r1}-U_{r2}) &{} r_5 > FAD\\ \end{array}\right. \end{aligned}$$
  In Eq. (20), $FAD=0.2$, and *W* is a binary solution (0 or 1) that corresponded to random solutions. If the random solution is less than 0.2, it converted to 0 while the random solution becomes 1 when the solutions are greater than 0.2. The symbol $r\in [0,1]$ represents a random number. $r_1$ and $r_2$ are the random index of the prey.
- Marine memory: This is the main feature of the marine predators and it helps in catching the optimal solution very fast and avoid local solutions. Faramarzi et al.^37^ implement this feature via saving the previous best solutions of a prior iteration, and compared with the current ones; the solutions are modified based on the best one during the comparison stage.

#### Fractional-order Marine Predators Algorithm (FO-MPA)

Recently, a combination between the fractional calculus tool and the meta-heuristics opens new doors in providing robust and reliable variants^41^. For this motivation, we utilize the FC concept with the MPA algorithm to boost the second step of the standard version of the algorithm. Hence, the FC memory is applied during updating the prey locating in the second step of the algorithm to enhance the exploitation stage. Moreover, the $R_B$ parameter has been changed to depend on weibull distribution as described below.

**Figure 2:**
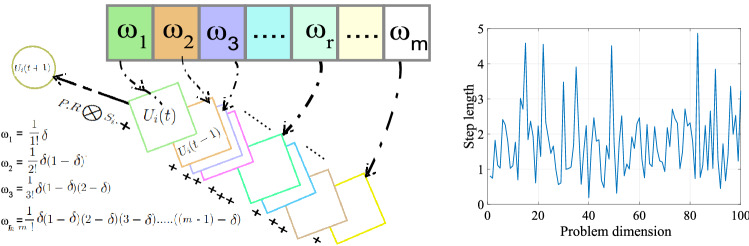
Memory FC prospective concept (left) and weibull distribution (right).

- *First: prey motion based on FC* the motion of the prey of Eq. (15) can be reformulated to meet the special case of GL definition of Eq. (9) as follows.
  21 $$\begin{aligned} U_i(t+1)-U_i(t)=P.R\bigotimes S_i \end{aligned}$$
  For general case based on the FC definition, the Eq. (22) can be written as follows:
  22 $$\begin{aligned} D ^{\delta } \left[ U_{i}(t+1)\right] =P.R\bigotimes S_i \end{aligned}$$
  By using the discrete form of GL definition of Eq. (8) at $T = 1$, the expression of Eq. (22) can be written as follows:
  23 $$D^{\delta } \left[ {U_{i} (t + 1)} \right] = U_{i} (t + 1) + \sum\limits_{{k = 1}}^{m} {\frac{{( - 1)^{k} \Gamma (\delta + 1)U_{i} (t + 1 - k)}}{{\Gamma (k + 1)\Gamma (\delta - k + 1)}}} = P \cdot R \otimes S_{i} .$$
  By taking into account the early mentioned relation in Eq. (23), the general formulation for the solutions of FO-MPA based on FC memory perspective can be written as follows:
  24 $$\begin{aligned} \begin{aligned} U(t+1)_{i}= - \sum _{k=1}^{m} \frac{(-1)^k\Gamma (\delta +1)U_{i}(t+1-k)}{\Gamma (k+1)\Gamma (\delta -k+1)} + P.R\bigotimes S_i. \end{aligned} \end{aligned}$$
  After checking the previous formula, it can be detected that the motion of the prey becomes based on some terms from the previous solutions with a length of (m), as depicted in Fig. 2 (left). With accounting the first four previous events ($m=4$) from the memory data with derivative order $\delta$, the position of prey can be modified as follow;
  25 $$\begin{aligned} \begin{aligned} U_{i}(t+1)&= \frac{1}{1!} \delta U_{i}(t)+ \frac{1}{2!}\delta (1-\delta ) U_{i}(t-1)+ \frac{1}{3!}\delta (1-\delta )(2-\delta ) U_{i}(t-2)\\&\quad + \frac{1}{4!}\delta (1-\delta )(2-\delta )(3-\delta ) U_{i}(t-3) + P.R\bigotimes S_i. \end{aligned} \end{aligned}$$
- Second: Adjusting $R_B$ random parameter based on weibull distribution. For the exploration stage, the weibull distribution has been applied rather than Brownian to bost the performance of the predator in stage 2 and the prey velocity in stage 1 based on the following formula:
  26 $$\begin{aligned} WF(x)=\exp ^{\left( {\frac{x}{k}}\right) ^\zeta } \end{aligned}$$
  Where *k*, and $\zeta$ are the scale and shape parameters. The Weibull Distribution is a heavy-tied distribution which presented as in Fig. 2 (right). In the current work, the values of *k*, and $\zeta$ are set to 2, and 2, respectively.

Our proposed approach is called Inception Fractional-order Marine Predators Algorithm (IFM), where we combine Inception (I) with Fractional-order Marine Predators Algorithm (FO-MPA). The proposed IFM approach is summarized as follows:

1. Extracting deep features from Inception, where about 51 K features were extracted.
2. Initialize solutions for the prey and predator. The prey follows Weibull distribution during discovering the search space to detect potential locations of its food.
3. The predator tries to catch the prey while the prey exploits the locations of its food. The predator uses the Weibull distribution to improve the exploration capability. Meanwhile, the prey moves effectively based on its memory for the previous events to catch its food, as presented in Eq. (24).
4. Finally, the predator follows the levy flight distribution to exploit its prey location. all above stages are repeated until the termination criteria is satisfied.

**Figure 3:**
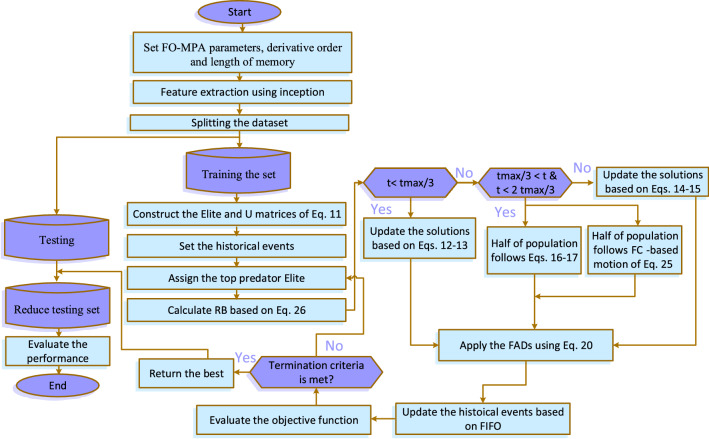
Proposed COVID-19 X-ray classification.

The memory terms of the prey are updated at the end of each iteration based on first in first out concept. Figure 3 illustrates the structure of the proposed IMF approach.

### Dataset description

In this paper, we used two different datasets. The first one, dataset 1 was collected by Joseph Paul Cohen and Paul Morrison and Lan Dao^42^, where some COVID-19 images were collected by an Italian Cardiothoracic radiologist. Negative COVID-19 images were collected from another Chest X-ray Kaggle published dataset^43^. The whole dataset contains around 200 COVID-19 positive images and 1675 negative COVID19 images. The data was collected mainly from retrospective cohorts of pediatric patients from Guangzhou Women and Children’s medical center. While the second dataset, dataset 2 was collected by a team of researchers from Qatar University in Qatar and the University of Dhaka in Bangladesh along with collaborators from Pakistan and Malaysia medical doctors^44^. Moreover, other COVID-19 positive images were added by the Italian Society of Medical and Interventional Radiology (SIRM) COVID-19 Database^45^. This dataset consists of 219 COVID-19 positive images and 1341 negative COVID-19 images.

These datasets contain hundreds of frontal view X-rays and considered the largest public resource for COVID-19 image data. They were manually aggregated from various web based repositories into a machine learning (ML) friendly format with accompanying data loader code. They were also collected frontal and lateral view imagery and metadata such as the time since first symptoms, intensive care unit (ICU) status, survival status, intubation status, or hospital location.

Both datasets shared some characteristics regarding the collecting sources. For both datasets, the Covid19 images were collected from patients with ages ranging from 40-84 from both genders. It is also noted that both datasets contain a small number of positive COVID-19 images, and up to our knowledge, there is no other sufficient available published dataset for COVID-19. Table 2 shows some samples from two datasets.

**Table 2:**
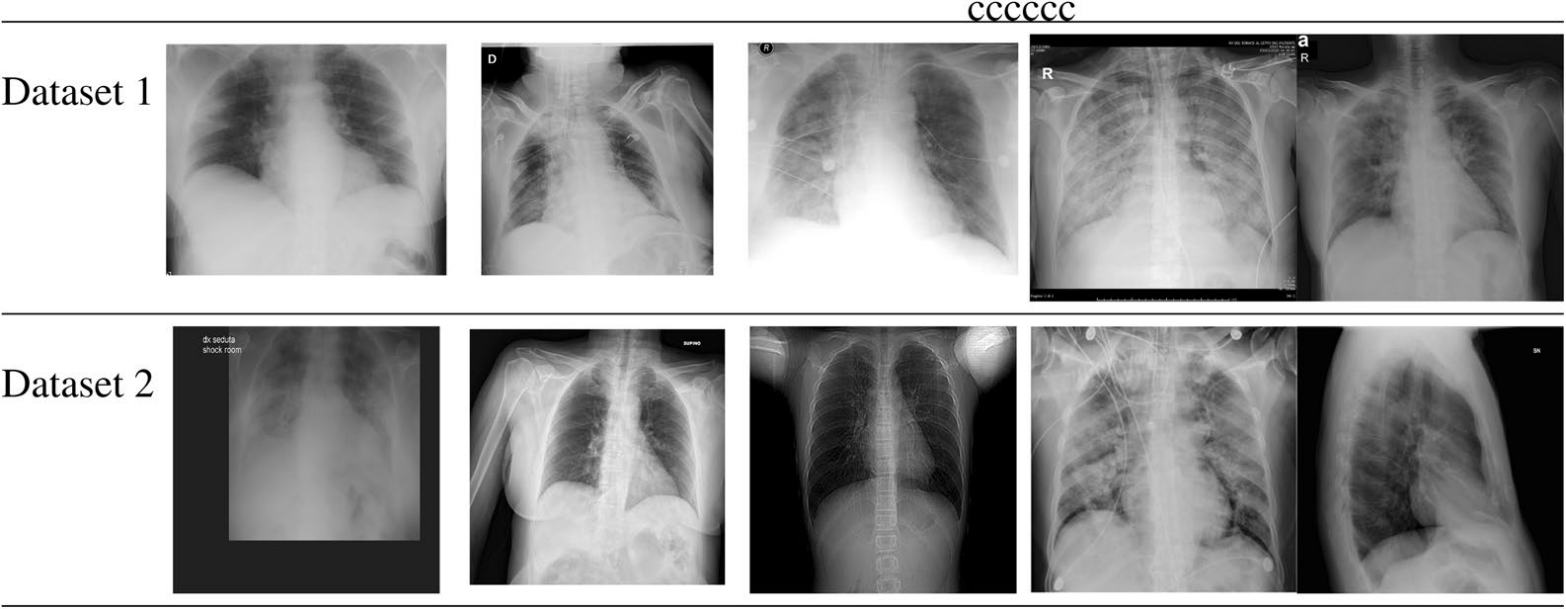
Samples from COVID-19 dataset 1^42^ and dataset 2^44^.

Table 2 depicts the variation in morphology of the image, lighting, structure, black spaces, shape, and zoom level among the same dataset, as well as with the other dataset.

### Validation metrics

To evaluate the performance of the proposed model, we computed the average of both best values and the worst values (Max) as well as STD and computational time for selecting features. The accuracy measure is used in the classification phase. The definitions of these measures are as follows:

27 $$\begin{aligned}&Accuracy = \frac{\text {TP} + \text {TN}}{\text {TP} + \text {TN} + \text {FP} + \text {FN}} \end{aligned}$$

28 $$\begin{aligned}&Sensitivity = \frac{\text {TP}}{\text{ TP } + \text {FN}}\end{aligned}$$

29 $$\begin{aligned}&Specificity = \frac{\text {TN}}{\text {TN} + \text {FP}}\end{aligned}$$

30 $$\begin{aligned}&F_{Score} = 2\times \frac{\text {Specificity} \times \text {Sensitivity}}{\text {Specificity} + \text {Sensitivity}} \end{aligned}$$

where “TP” (true positives) refers to the positive COVID-19 images that were correctly labeled by the classifier, while “TN” (true negatives) is the negative COVID-19 images that were correctly labeled by the classifier. “FP” (false positives) are the positive COVID-19 images that were incorrectly labeled as negative COVID-19, while “FN” (false negatives) are the negative COVID-19 images that were mislabeled as positive COVID-19 images.

- Best accuracy:
  31 $$\begin{aligned} Best_{acc} = \max _{1 \le i\le {r}} Accuracy \end{aligned}$$
- Best fitness value:
  32 $$\begin{aligned} Best_{Fit_i} = \min _{1 \le i\le r} Fit_i \end{aligned}$$
- Worst fitness value:
  33 $$\begin{aligned} Max_{Fit_i} = \max _{1 \le i\le r} Fit_i \end{aligned}$$
- Average of fitness value:
  34 $$\begin{aligned} \mu = \frac{1}{r} \sum _{i=1}^N Fit_i \end{aligned}$$
- Standard deviation of fitness value
  35 $$\begin{aligned} STD = \sqrt{\frac{1}{r-1}\sum _{i=1}^{r}{(Fit_i-\mu )^2}} \end{aligned}$$
  where *r* is the run numbers. $Fit_i$ denotes a fitness function value.

### Implementation environment

Convolutional neural networks were implemented in Python 3 under Google Colaboratory^46^, commonly referred to as “Google Colab,” which is a research project for prototyping machine learning models on powerful hardware options such as GPUs and TPUs. In this paper, we used TPUs for powerful computation, which is more appropriate for CNN. The model was developed using Keras library^47^ with Tensorflow backend^48^.

## Results and discussion

### Performance of the proposed approach

As Inception examines all X-ray images over and over again in each epoch during the training, these rapid ups and downs are slowly minimized in the later part of the training. After feature extraction, we applied FO-MPA to select the most significant features.

In this subsection, the results of FO-MPA are compared against most popular and recent feature selection algorithms, such as Whale Optimization Algorithm (WOA)^49^, Henry Gas Solubility optimization (HGSO)^50^, Sine cosine Algorithm (SCA), Slime Mould Algorithm (SMA)^51^, Particle Swarm Optimization (PSO), Grey Wolf Optimization (GWO)^52^, Harris Hawks Optimization (HHO)^53^, Genetic Algorithm (GA), and basic MPA. In this paper, each feature selection algorithm were exposed to select the produced feature vector from Inception aiming at selecting only the most relevant features. The parameters of each algorithm are set according to the default values. They shared some parameters, such as the total number of iterations and the number of agents which were set to 20 and 15, respectively. For fair comparison, each algorithms was performed (run) 25 times to produce statistically stable results.The results are listed in Tables 3 and 4. Table 3 shows the numerical results of the feature selection phase for both datasets. Four measures for the proposed method and the compared algorithms are listed. As seen in Table 3, on Dataset 1, the FO-MPA outperformed the other algorithms in the mean of fitness value as it achieved the smallest average fitness function value followed by SMA, HHO, HGSO, SCA, BGWO, MPA, and BPSO, respectively whereas, the SGA and WOA showed the worst results. The results of max measure (as in Eq. (33)), showed that FO-MPA also achieved the best value of the fitness function compared to others. SMA is on the second place, While HGSO, SCA, and HHO came in the third to fifth place, respectively. According to the best measure, the FO-MPA performed similarly to the HHO algorithm, followed by SMA, HGSO, and SCA, respectively. Although the performance of the MPA and bGWO was slightly similar, the performance of SGA and WOA were the worst in both max and min measures. Generally, the most stable algorithms On dataset 1 are WOA, SCA, HGSO, FO-MPA, and SGA, respectively. However, WOA showed the worst performances in these measures; which means that if it is run in the same conditions several times, the same results will be obtained. For Dataset 2, FO-MPA showed acceptable (not the best) performance, as it achieved slightly similar results to the first and second ranked algorithm (i.e., MPA and SMA) on mean, best, max, and STD measures. Also, WOA algorithm showed good results in all measures, unlike dataset 1, which can conclude that no algorithm can solve all kinds of problems. Whereas, the worst algorithm was BPSO.

**Table 3:** Results of the feature selection phase based on fitness function. Highest results are in bold.

	Dataset 1				Dataset 2			
	Mean	STD	$Best_{fi}$	Max	Mean	STD	$Best_{fi}$	Max
SMA	0.0388	0.0054	0.0316	0.0471	0.0212	**0**.**0025**	0.0166	0.0257
FO-MPA	**0**.**0361**	0.0044	0.0290	**0**.**0419**	0.0249	0.0039	0.0193	0.0316
MPA	0.1362	0.0092	0.1256	0.1515	**0**.**0189**	0.0027	**0**.**0161**	**0**.**0247**
HHO	0.0409	0.0112	**0**.**0285**	0.0699	0.1124	0.0127	0.0894	0.1328
HGSO	0.0428	0.0038	0.0373	0.0472	0.0240	0.0034	0.0192	0.0316
WOA	0.5246	**0**.**0024**	0.5246	0.5246	0.0218	0.0034	0.0166	0.0268
SCA	0.0441	0.0026	0.0398	0.0492	0.0230	0.0030	0.0200	0.0306
bGWO	0.1300	0.0074	0.1202	0.1445	0.1570	0.0638	0.1087	0.3252
SGA	0.5050	0.0046	0.4982	0.5117	0.1135	0.0100	0.0995	0.1267
BPSO	0.2274	0.0068	0.2137	0.2362	0.4214	0.0074	0.4028	0.4298

**Figure 4:**
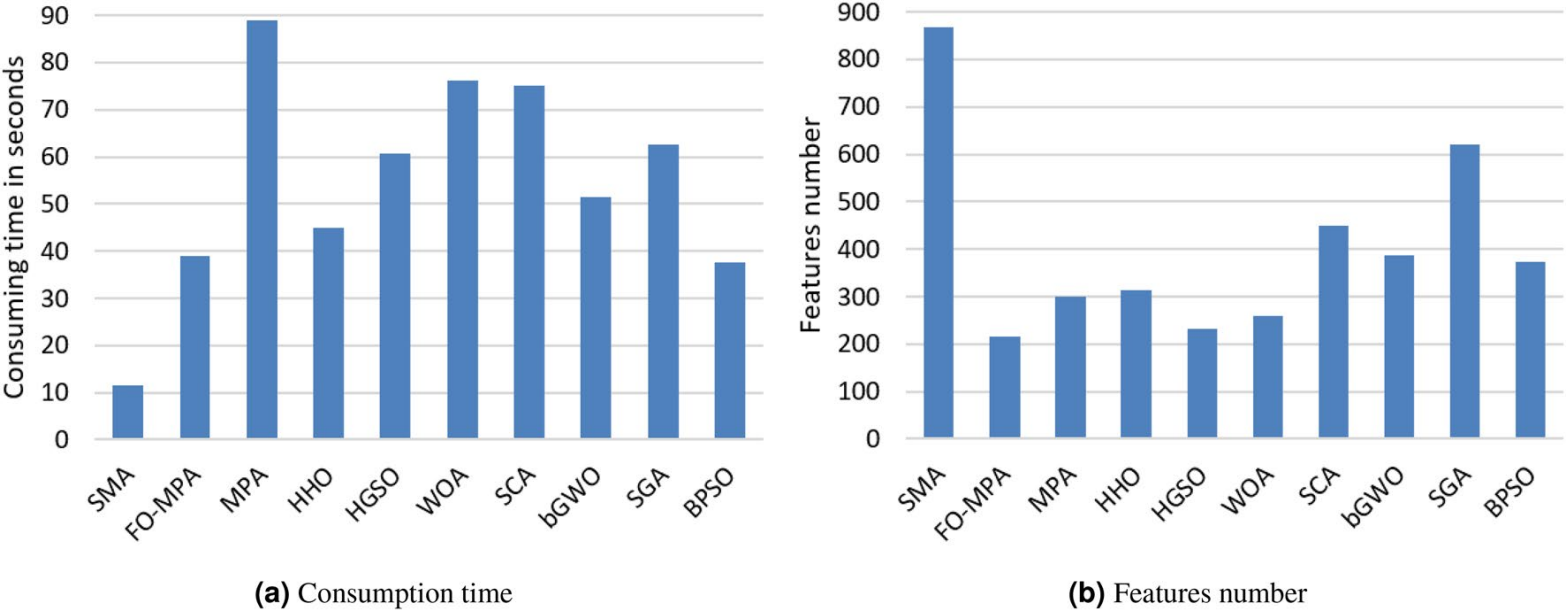
Average of the consuming time and the number of selected features in both datasets.

For more analysis of feature selection algorithms based on the number of selected features (S.F) and consuming time, Fig. 4 and Table 4 list these results for all algorithms. Regarding the consuming time as in Fig. 4a, the SMA was considered as the fastest algorithm among all algorithms followed by BPSO, FO-MPA, and HHO, respectively, while MPA was the slowest algorithm. Also, As seen in Fig. 4b, FO-MPA algorithm selected successfully fewer features than other algorithms, as it selected 130 and 86 features from Dataset 1 and Dataset 2, respectively. HGSO was ranked second with 146 and 87 selected features from Dataset 1 and Dataset 2, respectively. The largest features were selected by SMA and SGA, respectively.

**Figure 5:**
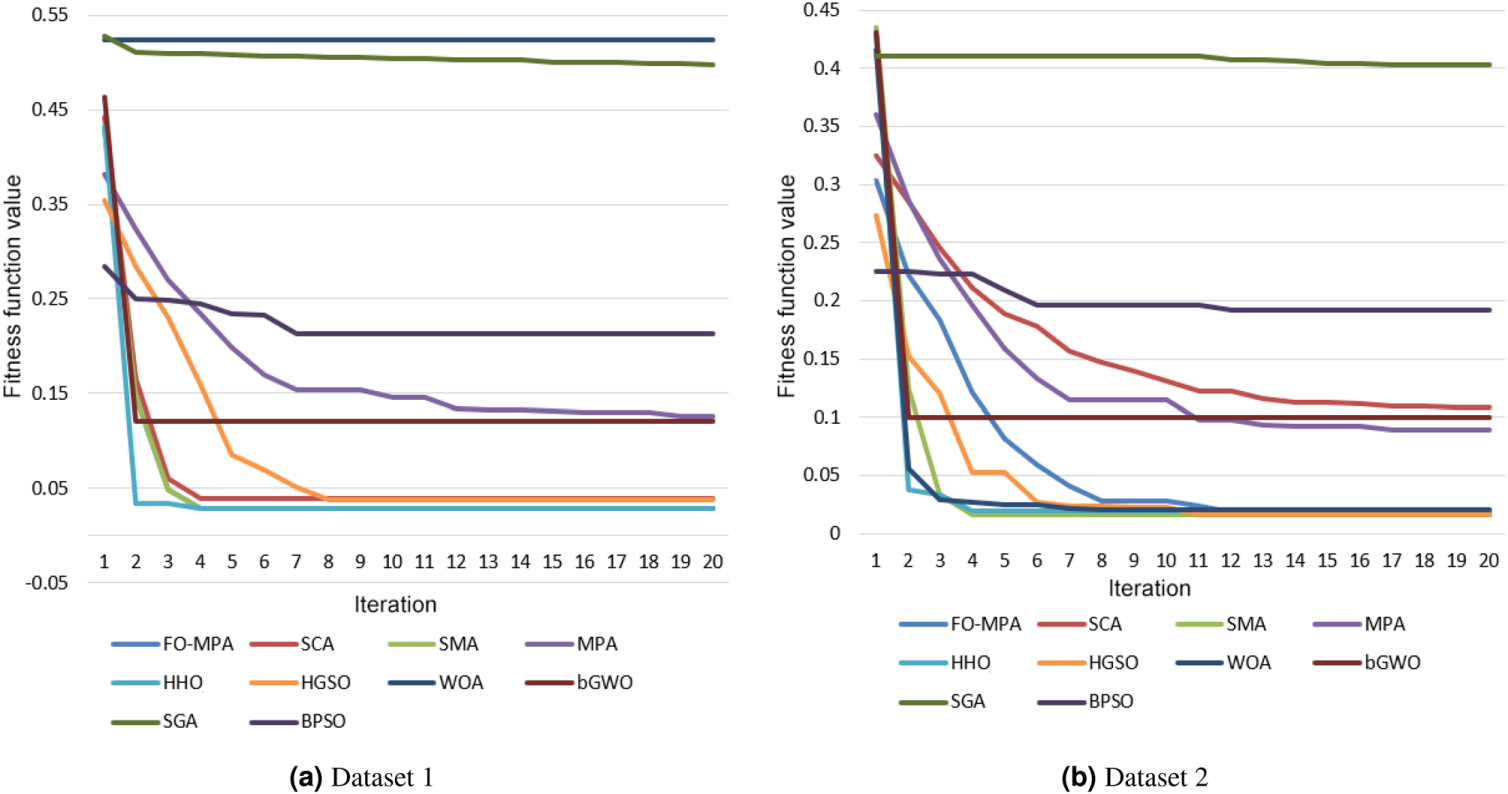
Convergence curves for both datasets.

The convergence behaviour of FO-MPA was evaluated over 25 independent runs and compared to other algorithms, where the x-axis and the y-axis represent the iterations and the fitness value, respectively. Figure 5 illustrates the convergence curves for FO-MPA and other algorithms in both datasets.

Figure 5, shows that FO-MPA shows an efficient and faster convergence than the other optimization algorithms on both datasets. Whereas, the slowest and the insufficient convergences were reported by both SGA and WOA in Dataset 1 and by SGA in Dataset 2.

To further analyze the proposed algorithm, we evaluate the selected features by FO-MPA by performing classification. In this experiment, the selected features by FO-MPA were classified using KNN. Table 4 show classification accuracy of FO-MPA compared to other feature selection algorithms, where the best, mean, and STD for classification accuracy were calculated for each one, besides time consumption and the number of selected features (SF). In Table 4, for Dataset 1, the proposed FO-MPA approach achieved the highest accuracy in the best and mean measures, as it reached 98.7%, and 97.2% of correctly classified samples, respectively. While, MPA, BPSO, SCA, and SGA obtained almost the same accuracy, followed by both bGWO, WOA, and SMA. The lowest accuracy was obtained by HGSO in both measures. Based on Standard Deviation measure (STD), the most stable algorithms were SCA, SGA, BPSO, and bGWO, respectively. Whereas, FO-MPA, MPA, HGSO, and WOA showed similar STD results. The HGSO also was ranked last. In Dataset 2, FO-MPA also is reported as the highest classification accuracy with the best and mean measures followed by the BPSO. The classification accuracy of MPA, WOA, SCA, and SGA are almost the same. Whereas the worst one was SMA algorithm. Besides, all algorithms showed the same statistical stability in STD measure, except for HHO and HGSO. Generally, the proposed FO-MPA approach showed satisfying performance in both the feature selection ratio and the classification rate. Moreover, from Table 4, it can be seen that the proposed FO-MPA provides better results in terms of F-Score, as it has the highest value in datatset1 and datatset2 which are 0.9821 and 0.99079, respectively.

**Table 4:** Performance of proposed approach.  Highest results are in bold.

Method	Dataset 1						Dataset 2					
	$Best_{acc}$	Mean	STD	Time	S.F	F-Score	$Best_{acc}$	Mean	STD	Time	S.F	F-Score
SMA	0.9569	0.9385	0.0107	**6**.**77**	430.12	0.97518	0.9808	0.9722	0.0054	**4**.**81**	436.70	0.98201
FO-MPA	**0**.**9877**	**0**.**9726**	0.0084	23.97	**129**.**50**	**0**.**98208**	**0**.**9968**	**0**.**9869**	0.0051	14.90	**86**.**00**	**0**.**99079**
MPA	0.9692	0.9508	0.0088	59.12	202.20	0.97183	0.9872	0.9812	0.0055	29.86	97.60	0.98502
HHO	0.9538	0.9295	0.0257	30.18	225.20	0.96014	0.9872	0.9690	0.0115	14.68	87.80	0.97552
HGSO	0.9385	0.9277	0.0087	31.24	146.10	0.9529	0.9840	0.9722	0.0114	29.34	87.30	0.97597
WOA	0.9508	0.9508	0.0080	58.17	158.40	0.97193	0.9904	0.9754	0.0096	18.05	99.90	0.97952
SCA	0.9569	0.9569	**0**.**0030**	59.91	358.20	0.97603	0.9872	0.9760	0.0071	15.13	92.50	0.99072
bGWO	0.9600	0.9492	0.0076	30.29	295.80	0.97364	0.9732	0.9808	0.0050	21.23	92.30	0.98535
SGA	0.9631	0.9560	0.0046	35.16	242.40	0.97213	0.9783	0.9840	**0**.**0029**	27.54	378.50	0.99065
BPSO	0.9600	0.9535	0.0068	19.79	187.00	0.97666	0.9904	0.9843	0.0051	17.70	185.40	0.98921

### Comparison with other CNN architectures

**Figure 6:**
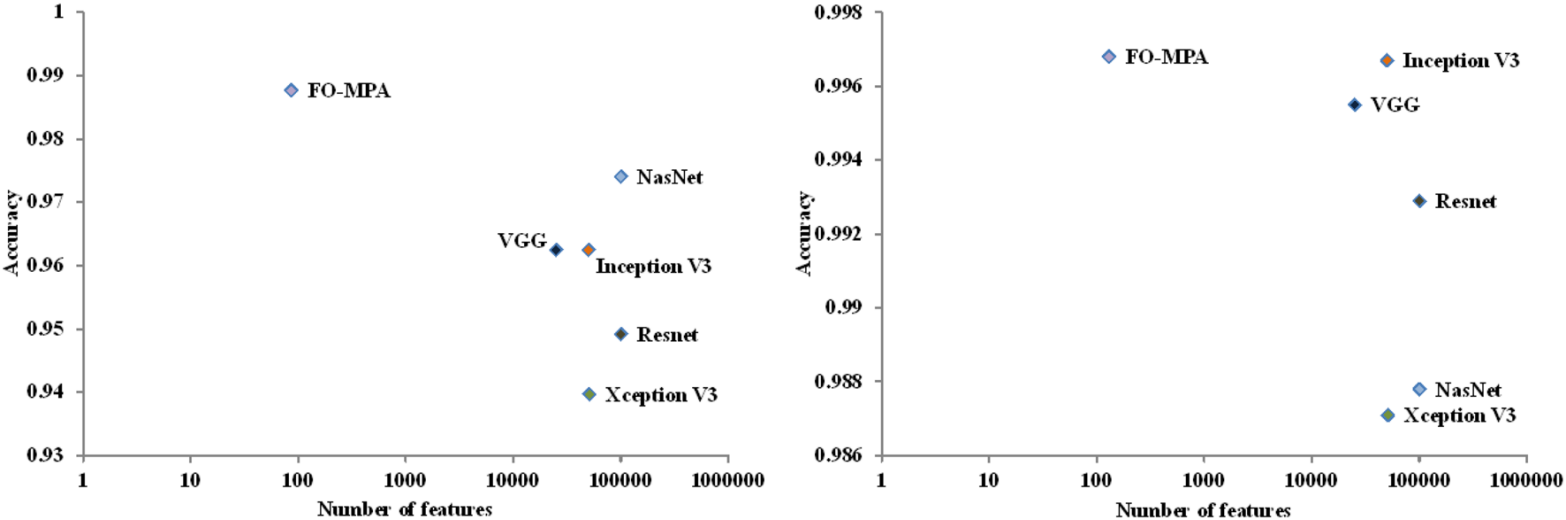
Number of extracted feature and classification accuracy by FO-MPA compared to other CNNs on dataset 1 (left) and on dataset 2 (right).

In this subsection, the performance of the proposed COVID-19 classification approach is compared to other CNN architectures. It noted that all produced feature vectors by CNNs used in this paper are at least bigger by more than 300 times compared to that produced by FO-MPA in terms of the size of the featureset. For example, as our input image has the shape $224 \times 224 \times 3$, Nasnet^26^ produces 487 K features, Resnet^25^ and Xception^29^ produce about 100 K features and Mobilenet^27^ produces 50 K features, while FO-MPA produces 130 and 86 features for both dataset1 and dataset 2, respectively. Figure 6 shows a comparison between our FO-MPA approach and other CNN architectures.

From Fig. 6 (left), for dataset 1, it can be seen that our proposed FO-MPA approach outperforms other CNN models like VGGNet, Xception, Inception, Mobilenet, Nasnet, and Resnet. It also shows that FO-MPA can select the smallest subset of features, which reflects positively on performance. Accordingly, that reflects on efficient usage of memory, and less resource consumption. On the second dataset, dataset 2 (Fig. 6, right), our approach still provides an overall accuracy of 99.68%, putting it first with a slight advantage over MobileNet (99.67 %).

#### Comparison with related works

**Figure 7:**
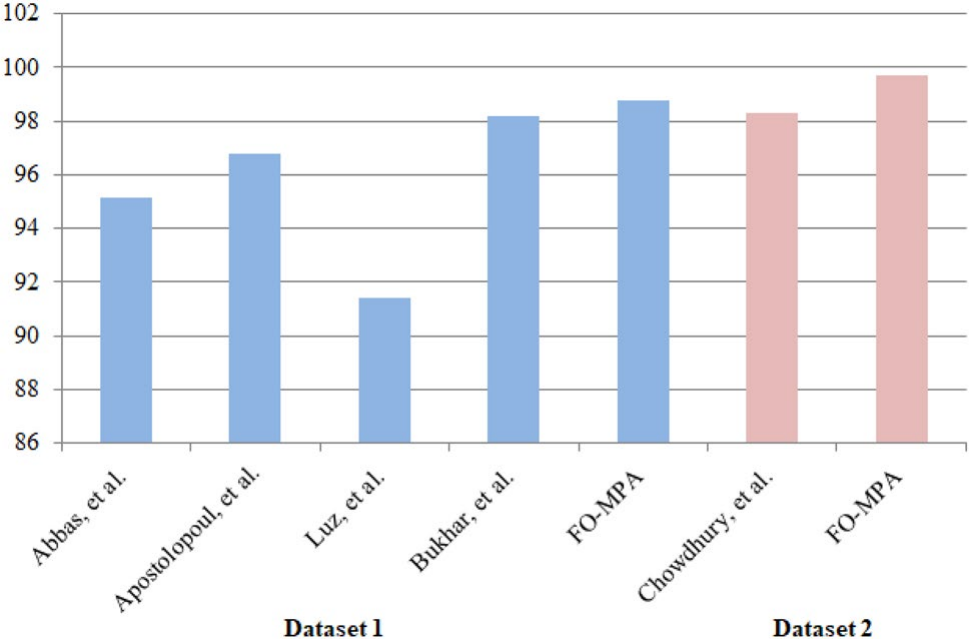
Comparison with other previous works using accuracy measure.

In this subsection, a comparison with relevant works is discussed. Figure 7 shows the most recent published works as in^54–57^ and^44^ on both dataset 1 and dataset 2. In^54^, AlexNet pre-trained network was used to extract deep features then applied PCA to select the best features by eliminating highly correlated features. Based on^54^, the later step reduces the memory requirements, and improve the efficiency of the framework. While^55^ used different CNN structures. However, it was clear that VGG19 and MobileNet achieved the best performance over other CNNs. Also, in^58^ a new CNN architecture called EfficientNet was proposed, where more blocks were added on top of the model after applying normalization of images pixels intensity to the range (0 to 1). Also, some image transformations were applied, such as rotation, horizontal flip, and scaling. In^57^, ResNet-50 CNN has been applied after applying horizontal flipping, random rotation, random zooming, random lighting, and random wrapping on raw images. As seen in Fig. 7, most works are pre-prints for two main reasons; COVID-19 is the most recent and trend topic; also, there are no sufficient datasets that can be used for reliable results. However, the proposed FO-MPA approach has an advantage in performance compared to other works. Also, all other works do not give further statistics about their model’s complexity and the number of featurset produced, unlike, our approach which extracts the most informative features (130 and 86 features for dataset 1 and dataset 2) that imply faster computation time and, accordingly, lower resource consumption. Compared to^59^ which is one of the most recent published works on X-ray COVID-19, a combination between You Only Look Once (YOLO) which is basically a real time object detection system and DarkNet as a classifier was proposed. They achieved 98.08 % and 96.51 % of accuracy and F-Score, respectively compared to our approach with 98.77 % and 98.2% for accuracy and F-Score, respectively. While no feature selection was applied to select best features or to reduce model complexity.

## Discussion

The proposed IMF approach successfully achieves two important targets, selecting small feature numbers with high accuracy. Therefore, reducing the size of the feature from about 51 K as extracted by deep neural networks (Inception) to be 128.5 and 86 in dataset 1 and dataset 2, respectively, after applying FO-MPA algorithm while increasing the general performance can be considered as a good achievement as a machine learning goal. Besides, the used statistical operations improve the performance of the FO-MPA algorithm because it supports the algorithm in selecting only the most important and relevant features. It also contributes to minimizing resource consumption which consequently, reduces the processing time.

In addition, the good results achieved by the FO-MPA against other algorithms can be seen as an advantage of FO-MPA, where a balancing between exploration and exploitation stages and escaping from local optima were achieved. As a result, the obtained outcomes outperformed previous works in terms of the model’s general performance measure.

Furthermore, using few hundreds of images to build then train Inception is considered challenging because deep neural networks need large images numbers to work efficiently and produce efficient features. However, the proposed IMF approach achieved the best results among the compared algorithms in least time. One of the main disadvantages of our approach is that it’s built basically within two different environments. The first one is based on Python, where the deep neural network architecture (Inception) was built and the feature extraction part was performed. The second one is based on Matlab, where the feature selection part (FO-MPA algorithm) was performed. So, there might be sometimes some conflict issues regarding the features vector file types or issues related to storage capacity and file transferring.

## Conclusion

Computational image analysis techniques play a vital role in disease treatment and diagnosis. Taking into consideration the current spread of COVID-19, we believe that these techniques can be applied as a computer-aided tool for diagnosing this virus. Therefore, in this paper, we propose a hybrid classification approach of COVID-19. It based on using a deep convolutional neural network (Inception) for extracting features from COVID-19 images, then filtering the resulting features using Marine Predators Algorithm (MPA), enhanced by fractional-order calculus(FO).

The proposed IMF approach is employed to select only relevant and eliminate unnecessary features. Extensive evaluation experiments had been carried out with a collection of two public X-ray images datasets. Extensive comparisons had been implemented to compare the FO-MPA with several feature selection algorithms, including SMA, HHO, HGSO, WOA, SCA, bGWO, SGA, BPSO, besides the classic MPA. The results showed that the proposed approach showed better performances in both classification accuracy and the number of extracted features that positively affect resource consumption and storage efficiency. The results are the best achieved compared to other CNN architectures and all published works in the same datasets.

According to the promising results of the proposed model, that combines CNN as a feature extractor and FO-MPA as a feature selector could be useful and might be successful in being applied in other image classification tasks.

## Data Availability

All data used in this paper is available online in the repository, [https://github.com/ieee8023/covid-chestxray-dataset], [https://stanfordmlgroup.github.io/projects/chexnet], [https://www.kaggle.com/paultimothymooney/chest-xray-pneumonia] and [https://www.sirm.org/en/category/articles/covid-19-database/]. The code of the proposed approach is also available via the following link [https://drive.google.com/file/d/1-oK-eeEgdCMCnykH364IkAK3opmqa9Rvasx/view?usp=sharing].
